# Analysis of Lignan Content and Rhizosphere Microbial Diversity of *Schisandra chinensis* (Turcz.) Baill. Resources

**DOI:** 10.3390/life14080946

**Published:** 2024-07-28

**Authors:** Yanli Wang, Yiming Yang, Changyu Li, Yingxue Liu, Shutian Fan, Yiping Yan, Taiping Tian, Jiaqi Li, Yue Wang, Hongyan Qin, Baoxiang Zhang, Wenpeng Lu, Peilei Xu

**Affiliations:** Institute of Special Animal and Plant Sciences, Chinese Academy of Agricultural Sciences, Changchun 130112, China; wangyanli@caas.cn (Y.W.); yangyiming@caas.cn (Y.Y.); lichangyu@caas.cn (C.L.); liuyingxue@caas.cn (Y.L.); fanshutian@caas.cn (S.F.); 82101225211@caas.cn (Y.Y.); 82101235243@caas.cn (T.T.); lijiaqi@caas.cn (J.L.); wangyue05@caas.cn (Y.W.); qinhongyan@caas.cn (H.Q.); zhangbaoxiang@caas.cn (B.Z.)

**Keywords:** *Schisandra chinensis*, lignans, rhizosphere microorganism, microbial diversity

## Abstract

Genetic and environmental factors influence the growth and quality of medicinal plants. In recent years, rhizosphere microorganisms have also emerged as significant factors affecting the quality of medicinal plants. This study aimed to identify *Schisandra* resources with high lignan content and analyze the microbial diversity of the rhizosphere soil. High-performance liquid chromatography was used to measure the lignan content in nine *Schisandra* fruits. High-throughput sequencing was used to analyze the 16S rDNA sequences of rhizosphere bacteria to identify bacterial species diversity. The total lignan content of the nine *Schisandra* resources ranged from 9.726 mg/g to 14.031 mg/g, with ZJ27 having the highest content and ZJ25 the lowest. Among the six lignan components, Schisandrol A had the highest content, ranging from 5.133 mg/g to 6.345 mg/g, with a significant difference between ZJ25, ZJ27, and other resources (*p* < 0.05). Schizandrin C had the lowest content, ranging from 0.062 mg/g to 0.419 mg/g, with more significant differences among the resources. A total of 903,933 sequences were obtained from the rhizosphere soil of the nine *Schisandra* resources, clustered into 10,437 OTUs at a 97% similarity level. The dominant bacterial phyla were *Actinobacteriota*, *Acidobacteriota*, *Proteobacteria*, *Chloroflexi*, *Gemmatimonadota*, and *Verrucomicrobiota*. The dominant bacterial genera were *Candidatus_Udaeobacter*, *Candidatus_Solibacter*, *RB41*, *Bradyrhizobium*, *Gaiella*, and *Arthrobacter*. ZJ27 is the *Schisandra* resource with the highest lignan content, and the rhizosphere bacteria of *Schisandra* are rich in diversity. Schisandra B is negatively correlated with *Bryobacter*, *Candidatus_Solibacter*, and unnamed genera of *Gaiellales*.

## 1. Introduction

*Schisandra chinensis* (Turcz.) Bail. (*S. chinensis*) is a member of the genus *Schisandra* within the *magnolia* family. It is primarily distributed in the Jilin, Liaoning, Heilongjiang, Neimenggu, Hebei, Shanxi, Ningxia, Gansu, and Shandong provinces of China, as well as in Russia, Korea, Japan, and other East Asian countries [1,2,3]. The fruit of *S. chinensis* is known for its five distinct flavors: sweet, bitter, spicy, salty, and sour [4]. As a traditional Chinese medicine, *S. chinensis* is widely used to treat conditions such as cough, insomnia, night sweats, liver disease, and kidney disease [5,6]. Modern medical research has confirmed that *S. chinensis* contains numerous active ingredients, including lignans, triterpenes, phenolic acids, flavonoids, essential oils, and polysaccharides [7]. These components have been found to exhibit therapeutic effects such as hepatoprotection, neuroprotection, cardiovascular protection, blood sugar and lipid regulation, and anti-cancer properties [7].

*Schisandra* lignans, also known as dibenzocyclooctadiene lignans, are a class of natural compounds found in *S. chinensis* [3]. Currently, more than 50 types of Schisandra lignans have been isolated, with schisandrol A, schisandrol B, schisantherin A, schisandrin A, schisandrin B, and schisandrin C being the primary lignan components [8,9,10]. Lignans are present in the seeds, roots, stems, leaves, and fruit, with the highest concentration typically found in the fruit [11].

Numerous clinical studies have confirmed that various lignans possess distinct functionalities [12,13]. Schisandrol A exhibits effects such as reducing neuroinflammation, acting as an antidepressant, inhibiting pulmonary fibrosis, and protecting the heart [14,15,16]. Schisandrol B has been shown to inhibit the proliferation of cancer cells, prevent diabetic osteoporosis, and protect the liver [17,18,19]. Schizantherin A can relieve fatigue, improve learning and memory in mice, and serve as a potential drug for oxidative stress-related cognitive dysfunction [20]. Schisandrin A has therapeutic effects on enteritis, osteoarthritis, and mastitis, and it can also improve the sequelae of ischemic brain injury [21,22,23]. Schisandrin B, with its antioxidant and anti-inflammatory properties, plays an important role in improving metabolism-related diseases and repairing the nervous system [24,25,26]. Schisandrin C can inhibit the activity of pancreatic enzymes associated with lipolysis, thereby reducing fat accumulation, and it can inhibit hepatitis B virus replication, making it a potential raw material for weight loss foods and antiviral drugs [27]. Schisandrin A, B, and C can inhibit the proliferation of Propionibacterium acnes, making them potential pharmaceutical ingredients for acne treatment [28]. With the in-depth study of *S. chinensis*, more beneficial characteristics have been discovered. In recent years, it has been widely used as an effective additive in medicine, cosmetics, and health care products. Additionally, it can serve as a functional food ingredient with a unique flavor in tea, beverages, jams, and seasonings [4,29]. As the yield of *S. chinensis* increases, one effective approach to maximize the benefits of *Schisandra* cultivation is through breeding superior strains and enhancing the quality of the plant.

In general, the lignan content in *S. chinensis* depends on the cultivation environment, the maturity of the fruit, and the harvest season. For most plants, there are also differences in quality traits between different varieties [30]. The growth environment includes sunlight, temperature, water, fertilizer, and soil microorganisms [31]. Recently, more research has shown that rhizosphere microorganisms can affect the absorption of nutrients, thereby influencing plant growth and development. Joseph Edwards et al. conducted a study and discovered archaea involved in methane cycling in rice rhizosphere soil; they also found that both soil source and genotype influenced the microbial composition of the rice rhizosphere [32]. Jin Xu et al. identified 11 bacterial genera beneficial to *Citrus* growth and health, including *Pseudomonas*, *Agrobacterium*, *Cupriavidus*, *Bradyrhizobium*, *Rhizobium*, *Mesorhizobium*, *Burkholderia*, *Cellvibrio*, *Sphingomonas*, *Variovorax*, and *Paraburkholderia* [33]. Juan E. Perez-Jaramillo et al. found that rhizosphere microbial diversity is related to specific root length, with a higher specific root length correlating with a higher relative abundance of Bacteroidetes [34]. Some studies have shown a close relationship between rhizosphere microbial diversity and the growth, quality, and health of *ginseng* and *Astragalus* [35,36,37].

In this study, we assessed the fruit characteristics and primary lignan content of nine *Schisandra* resources stored in the resource nursery. Additionally, we analyzed the rhizosphere soil microbial diversity of these nine resources to identify microorganisms associated with the growth, nutrition, and fruit quality of *Schisandra*. This research lays a foundation for enhancing the medicinal quality of *Schisandra* and breeding superior varieties.

## 2. Materials and Methods

### 2.1. Plant Material Used in the Experiment

**Figure 1:**
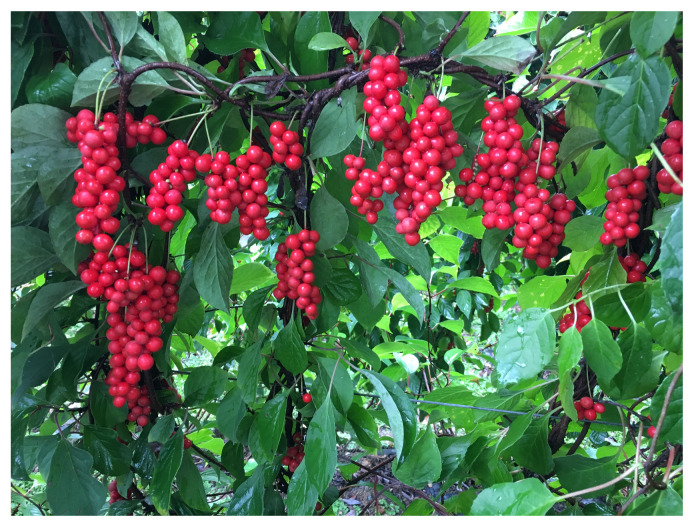
*Schisandra chinensis* at harvest time.

The *S. chinensis* used in the experiment were obtained from the *Schisandra* resource bank of the Institute of Special Animal and Plant Sciences, Chinese Academy of Agricultural Sciences (Figure 1). The region, with an average altitude of 194 m, experiences a temperate continental monsoon climate characterized by concurrent rain and heat during the same season, ample sunshine, a short frost-free period, an annual precipitation of 550 mm, a minimum temperature of −32 °C, and a maximum temperature of 35 °C.

The soil characteristics (Table 1) are as follows: pH of 5.31, organic matter content of 3.27%, total phosphorus of 807.06 mg/kg, total potassium of 18,441.82 mg/kg, total nitrogen of 1922.6 mg/kg, alkaline hydrolysable nitrogen of 164.02 mg/kg, available phosphorus of 142.57 mg/kg, and available potassium of 114.82 mg/kg.

### 2.2. Sampling Method

During the fruit ripening period, fruits (code: ZJ19-27) free from pests and diseases were collected and dried in an oven at 50°C. The dried fruits were then ground into a powder and sieved through a 60-mesh screen for the extraction and determination of lignan content [38,39]. Concurrently, the fibrous roots around the plant were collected and transported to the laboratory in an ice box. The soil adhering to the roots was carefully brushed onto sulfuric acid paper, collected into 2 mL centrifuge tubes, rapidly frozen in liquid nitrogen, and stored at −80°C for future analysis. Three replicates were prepared for each germplasm.

### 2.3. Fruit Characteristics Analysis

During the ripening period, three fruit bunches per resource (code: ZJ19-27) free from pests and diseases were collected. Seven phenotypic traits of nine accessions were observed. A vernier caliper (500-182-30, Mitutoyo, Kawasaki, Japan) was used to measure the length of fruit bunch (LB), the stalk length of the fruit bunch (LSB), the width of the fruit (WF), and the length of the fruit (LF). An electronic balance (BSA224S-CW, Sartorius, Beijing, China) was used to measure the number of fruits per fruit bunch (NFPB), the weight of the fruit bunch (FWB), the fresh weight of the fruit (FWF), and the dry weight of the fruit (DWF). SPSS 23.0 software was used for the statistical analysis of the traits.

### 2.4. Quantitative Analysis of Lignans

#### 2.4.1. Preparing the Standard Solution and Standard Curves

Lignans were extracted from *Schisandra chinensis* fruits using the method described by Wang et al. [38,39]. First, approximately 1 mg of each standard compound—schisandrol A, schisandrol B, schisantherin A, schisandrin A, schisandrin B, and schisandrin C—was measured. Methanol was then added to obtain standard solutions with concentrations of 0.200 mg/mL for schisandrol A, 0.286 mg/mL for schisandrol B, 0.248 mg/mL for schisantherin A, 0.250 mg/mL for schisandrin A, 0.286 mg/mL for schisandrol B, 0.248 mg/mL for schisantherin A, 0.250 mg/mL for schisandrin A, 0.258 mg/mL for schisandrin B, and 0.232 mg/mL for schisandrin C. Next, an Agilent 880975-902 SB-C18 Analytical 4.6 × 250 mm column was used for gradient elution with methanol (A) and water (B) as the mobile phase. The flow rate was set at 1.0 mL/min, the column temperature was maintained at 35 °C, the detection wavelength was 220 nm, and the sample injection volume was 10 μL. The gradient elution conditions are detailed in Table 2.

#### 2.4.2. Preparing and Testing Sample Solution, and Data Analysis

The sample powder (0.10 g) was accurately weighed and combined with 10 mL of methanol. The mixture was extracted in a water bath at 65 °C for 20 min, followed by ultrasonic extraction (320 W, 65 °C) for an additional 20 min [38,39]. It was then filtered using a 0.45 μm organic filter membrane and tested under the same conditions as described in Section 2.4.1. The difference in lignan content between different samples was analyzed using SPSS 23.0.

### 2.5. Rhizosphere Microbial Sequencing Analysis

#### 2.5.1. Total DNA Extraction, PCR Amplification, and Sequencing

Total microbial genomic DNA was extracted from rhizosphere soil samples of *S. chinensis* using the E.Z.N.A.^®^ Soil DNA Kit (Omega Bio-tek, Norcross, GA, USA) according to the manufacturer’s instructions. The quality of the extracted DNA was assessed by 1.0% agarose gel electrophoresis, and the concentration was measured using a NanoDrop^®^ ND-2000 spectrophotometer (Thermo Scientific Inc., Waltham, MA, USA). Qualified DNA was stored at −80 °C until further use.

The hypervariable V3V4 region of the bacterial 16S rRNA gene was amplified using the primer pairs 338F (5′-ACTCCTACGGGAGGCAGCAG-3′) and 806R (5′-GGACTACHVGGGTWTCTAAT-3′) [40] with an ABI GeneAmp^®^ 9700 PCR thermocycler (ABI, Oakland, CA, USA). The PCR products were then extracted from a 2% agarose gel, purified using the AxyPrep DNA Gel Extraction Kit (Axygen Biosciences, Union City, CA, USA) according to the manufacturer’s instructions, and quantified using a Quantus™ Fluorometer (Promega, Madison, WI, USA).

Purified amplicons were pooled in equimolar amounts and sequenced using paired-end sequencing on an Illumina MiSeq PE300 platform. The raw sequencing reads were deposited into the NCBI Sequence Read Archive (SRA) database (BioProject: PRJNA1126850). The sequencing service was commissioned by Majorbio Bio-Pharm Technology Co., Ltd. (Shanghai, China).

#### 2.5.2. Data Processing

Raw reads were demultiplexed using an in-house Perl script and quality-filtered with fastp (version 0.19.6) using the parameters: −q 20 and −u 3 [41]. The filtered reads were merged with FLASH (version 1.2.7) [42] using the following parameter: −m 10, −x 0.2, and −M 300. The optimized sequences were clustered into operational taxonomic units (OTUs) using UPARSE (version 7.1) [43,44] at a 97% sequence similarity threshold. The most abundant sequence for each OTU was selected as the representative sequence. Taxonomic annotation was performed using the RDP Classifier Bayesian algorithm [45]. Sequences representing OTUs at 97% similarity were annotated to obtain the taxonomic information for each OTU.

#### 2.5.3. Diversity Analysis

A bioinformatic analysis of the rhizosphere soil microbiota of *S. chinensis* was conducted using the Majorbio Cloud platform (https://cloud.majorbio.com) (accessed on 15 December 2020). Based on the OTU information, alpha diversity indices, including Chao1 richness, Shannon index, and Good’s coverage, were calculated using Mothur (version 1.30.1) [46]. Differences among the microbial communities in different samples were determined using R (version 3.3.1) and pheatmap (version 1.0.8). Similarity among the microbial communities in different samples was assessed through Non-metric Multidimensional Scaling (NMDS) based on Bray–Curtis dissimilarity, utilizing the Vegan (version 2.5.3) package. A Spearman Correlation Heatmap was generated using the pheatmap package to investigate the association between lignans and microorganisms.

## 3. Results

### 3.1. Analysis of Schisandra Chinensis Fruit Characteristics

**Figure 2:**
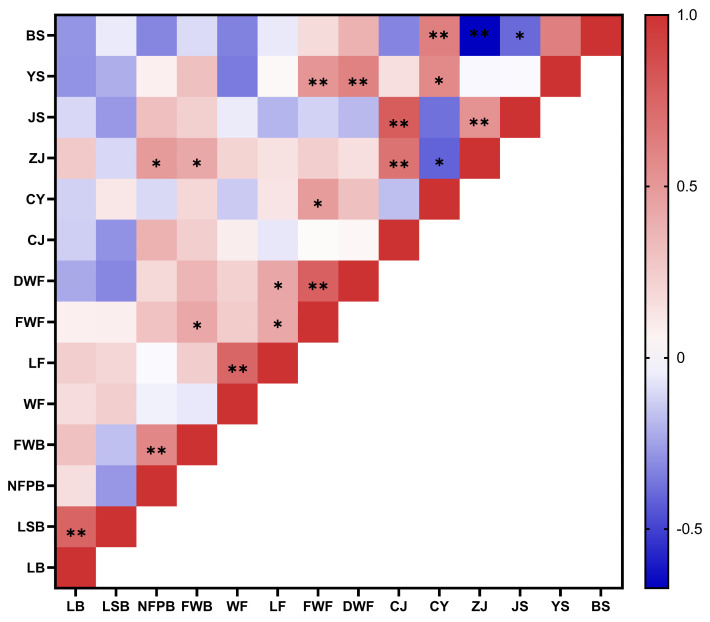
Correlations among eight fruit characteristics and six lignans. LB: length of fruit bunch; LSB: stalk length of fruit bunch; NFPB: number of fruits per fruit bunch; FWB: weight of fruit bunch; WF: width of fruit; LF: length of fruit; FWF: fresh weight of fruit; DWF: dry weight of fruit. CJ: Schisandrol A; CY: Schisandrol B; ZJ: Schisantherin A; JS: Schisandrin A; YS: Schisandrin B; BS: Schisandrin C. * indicates significant difference in *p* < 0.05 level; ** indicates significant difference in *p* < 0.01 level.

During the harvest season, we statistically analyzed the fruit characteristics of nine *Schisandra* resources. The traits examined included the length of the fruit bunch (LB), the stalk length of the fruit bunch (LSB), the number of fruits per fruit bunch (NFPB), the weight of the fruit bunch (FWB), the width of the fruit (WF), the length of the fruit (LF), the fresh weight of the fruit (FWF), and the dry weight of the fruit (DWF). The results are summarized in Table 3. We found that the average length of the fruit bunch was 9.878 cm, with the maximum value being 12.433 cm for the ZJ24 resource and the minimum value being 6.9 cm for the ZJ19 resource. The average stalk length of the fruit bunch was 4.033 cm, with the maximum value being 7.9 cm for ZJ24 and the minimum value being 2.567 cm for ZJ19. The average number of the fruits per fruit bunch was 24, with the maximum value being 32 for ZJ27 and the minimum value being 18.7 for the ZJ24. The average weight of the fruit bunch was 12.336 g, with the maximum value being 29.467 g for ZJ27 and the minimum value being 10.453 g for ZJ26. Among the nine resources, ZJ24 had the largest fruit size (width 14.017 mm, length 12.383 mm), while ZJ19 had the smallest fruit size (10.633 mm × 10.533 mm). In terms of variability, the stalk length of the fruit bunch (LSB) (RSD 8.65%), the number of fruits per fruit bunch (NFPB) (RSD 5.14%), and the weight of the fruit bunch (FWB) (RSD 6.78%) exhibited moderate variations (Table 3). The correlation analysis revealed a significant positive correlation between the length of the fruit bunch and the stalk length of the fruit bunch. There was also a significant positive correlation between the number of fruits per fruit bunch and the weight of the fruit bunch. Additionally, a significant positive correlation was observed between Schisandrin B and fruit weight (Figure 2).

### 3.2. Differential Analysis of Schisandra Lignan Component Content

SPSS 23.0 software was utilized to conduct a differential analysis of the content of six lignan components across 27 samples from nine different sources. The results revealed that the *Schisandra* lignan content in ZJ19 and ZJ27 was significantly higher than in other sources. Among the six lignan components, Schisandrol A had the highest content, while Schisandrin C had the lowest. The content of the other four lignan components varied among the different *Schisandra* fruit samples. The content of Schisandrol A across the nine sources ranged from 5.133 mg/g to 6.345 mg/g. At the *p* < 0.05 level, significant differences in Schisandrol A content were observed between ZJ25, ZJ27, and the other sources, while differences among the other sources were not significant. The content of Schisandrin C ranged from 0.419 mg/g to 0.062 mg/g. The difference in Schisandrin C content between ZJ21 and ZJ22, ZJ23, ZJ24, and ZJ25 was not significant, while the remaining differences were significant. Significant differences were also observed in the content of Schisandrol B, Schisandrin A, Schisandrin B, and Schisandrin C among different Schisandra resources (Table 4). The correlation analysis indicated a highly significant positive relationship between Schisandrol A and Schisantherin A, as well as Schisandrin A. Schisandrol B was significantly positively correlated with Schisandrin B and Schisandrin C, but significantly negatively correlated with Schisantherin A. Additionally, Schisandrin C was significantly negatively correlated with Schisantherin A and Schisandrin A (Figure 2).

### 3.3. Rhizosphere Microbial Community Composition of Schisandra

A 16S rDNA sequencing analysis was conducted on 27 samples, yielding a total of 903,933 valid sequences. Taxonomic classification analysis was performed on non-redundant sequences at a 97% similarity level, resulting in 10,437 operational taxonomic units (OTUs) belonging to 44 phyla, 255 families, and 1073 genera.

**Figure 3:**
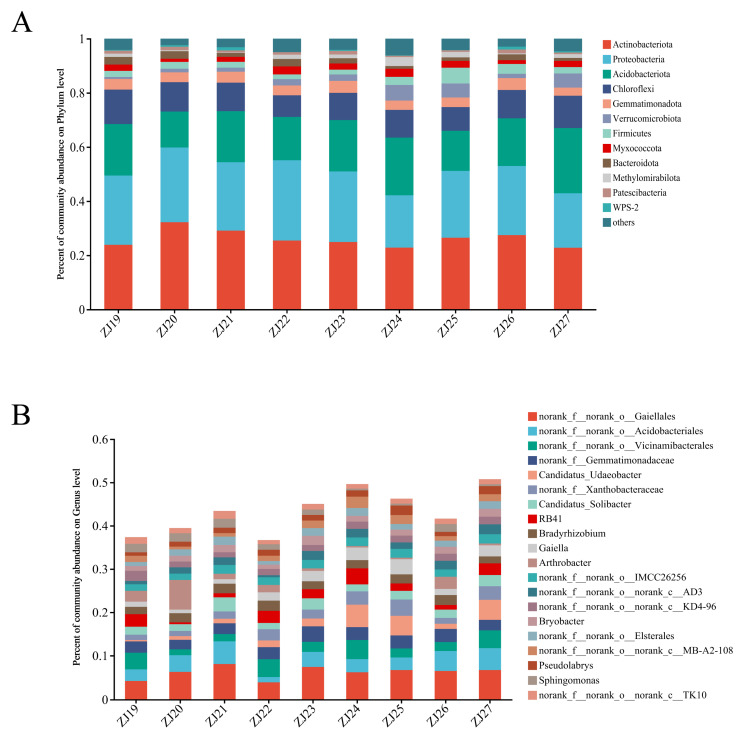
Community bar plot analysis of rhizosphere Bacterial of Different *S. chinensis* resources. (**A**) Community bar plot analysis at the phylum level of rhizosphere bacteria of different *S. Chinensis* resources. (**B**) Community bar plot analysis at the genus level of rhizosphere bacteria of different *S. chinensis* resources. ZJ19–ZJ27: Different *S. chinensis* fruit resources.

At the phylum level, species composition analysis identified twelve phyla with abundances greater than 1%. *Actinobacteriod* was the dominant phylum at 28%, followed by *Proteobacteria* at 26%, *Acidobacteriota* at 19%, and *Chloroflexi* at 10% (Figure 3A). The top 20 genera by abundance included *Candidatus_Udaeobacter*, *Candidatus_Solibacter*, *RB41*, *Bradyrhizobium*, *Gaiella*, and *Arthrobacter*. The abundance of *Arthrobacter* and *Sphingomonas* in samples ZJ24, ZJ25, and ZJ27 was extremely low, while the abundance of *Candidatus_Udaeobacter* was lowest in sample ZJ19, and *RB41* was lowest in sample ZJ20. *Arthrobacter* had the highest abundance in sample ZJ20 (Figure 3B).

### 3.4. Rhizosphere Soil Microbial Diversity of Schisandra

The microbial α-diversity indices for the samples show that the Shannon index of soil microbial communities associated with nine *Schisandra* resources ranged from 6.95 to 6.37, the Chao index ranged from 5789 to 3925, and the coverage index ranged from 0.97 to 0.96. This indicates that the rhizosphere microbial diversity and coverage trends in the nine *Schisandra* resources were relatively consistent, while there were significant differences in species richness. The rhizosphere microbial abundance was highest in resource ZJ22 and lowest in resources ZJ20 and ZJ21 (Table 5).

**Figure 4:**
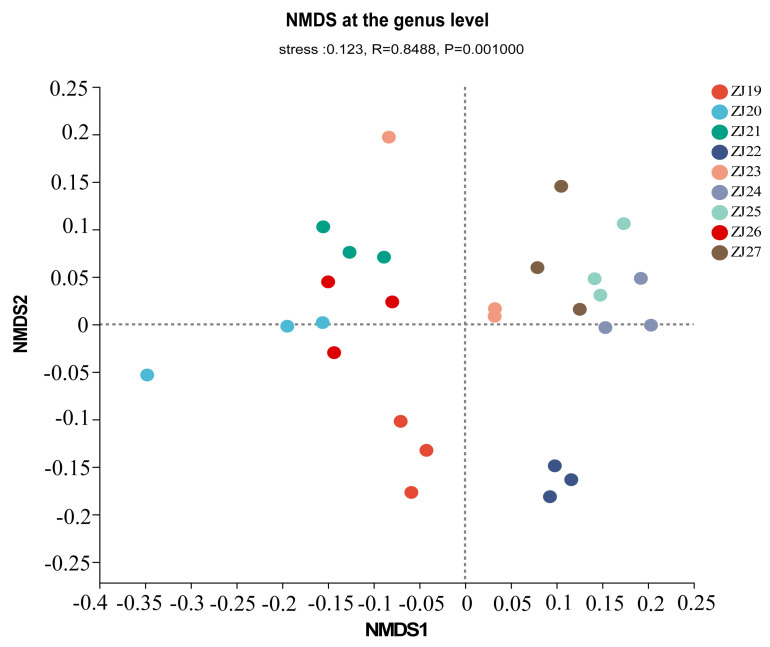
Beta diversity analysis of rhizosphere microbial community in different *S. chinensis* resources.

In the NMDS analysis, the similarity of bacterial composition at the genus level among different samples was examined. The results indicated an R value of 0.8488 and a *p* value of 0.001, suggesting significant differences in rhizosphere microbial composition among the nine *Schisandra* resources (Figure 4).

### 3.5. Correlation Analysis between Rhizosphere Microorganisms and Schisandra Lignans

**Figure 5:**
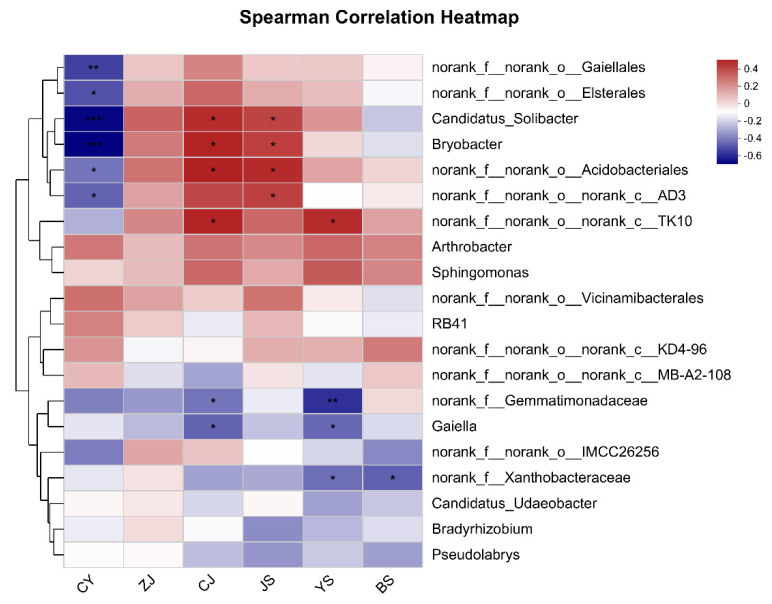
Correlation analysis between rhizosphere microorganisms and lignans in *S. chinensis.* CY: Schisandrol B; ZJ: Schisantherin A; CJ: Schisandrol A; JS: Schisandrin A; YS: Schisandrin B; BS: Schisandrin C; * means significant difference between samples (*p* < 0.05); ** means significant difference between samples (*p* ≤ 0.01); *** means significant difference between samples (*p* ≤ 0.001).

A correlation analysis was performed between the top 20 relative abundances of rhizosphere microorganisms and *Schisandra* lignans. The results indicated a significant correlation between 10 bacterial taxa and lignans. Schisandrol B showed a significant negative correlation with norank_f_norank_o_*Gaiellales*, norank_f_norank_o_*Elsterales*, *Candidatus_Solibacter*, *Bryobacter*, norank_f_norank_o_*Acidobacteriales*, and norank_f_norank_o_norank_c_*AD3*. Schisandrol A had significant positive correlations with *Candidatus_Solibacter*, *Bryobacter*, and norank_f_norank_o_*Acidobacteriales*, and a significant negative correlation with norank_f_*Gemmatimonadaceae*, *Gaiella*. Schisandrin A exhibited significant positive correlations with *Bryobacter*, *Candidatus_Solibacter*, norank_f_norank_o_*Acidobacteriales*, and norank_f_norank_o_norank_c_*AD3*. *Gaiella* was negatively correlated with all lignans and significantly negatively correlated with Schisandrol A and Schisandrin B (Figure 5).

### 3.6. Functional Prediction Analysis

**Figure 6:**
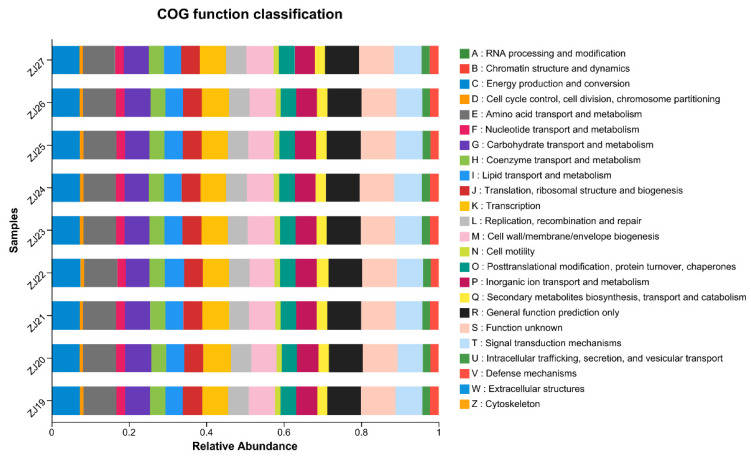
Prediction of rhizosphere microbial function; A~Z indicate function numbers and descriptions of COGs.

Using PICRUSt (Version 1.1.4 ) software, COG functional annotation was performed on the 16S rDNA sequences of rhizosphere microorganisms to predict their functional profiles. The results indicate that these microorganisms are primarily classified into 20 gene functional families (Figure 6). Approximately 41.5% of the genes are associated with metabolism, including amino acid transport and metabolism, energy production and conversion, carbohydrate transport and metabolism, and inorganic ion transport and metabolism. About 21% of the genes are related to cell formation and signaling, including signal transduction, cell wall, cell membrane synthesis, and cytoskeleton formation. Genes related to transcription, translation, and modification constitute approximately 20% of the total, while genes with unknown functions or poorly annotated results account for about 18%.

## 4. Discussion

Recent developments have introduced several *Schisandra* varieties, including ‘Yanzhihong’, ‘Hongzhenzhu’, ‘Yanhong’, and ‘Jinwuzi’, which are of significant importance for the cultivation and development of *S. chinensis* resource bank [47]. The quality of medicinal plants is influenced by factors such as genotypes, soil physicochemical properties, the climate, and soil microorganisms [48]. Currently, *S. chinensis* is primarily propagated through seedlings, resulting in variation in genotype and medicinal quality among plants [47]. To advance the *Schisandra* industry, it is crucial to breed superior varieties with desirable traits and to explore cultivation techniques that enhance quality and efficiency. In this study, we analyzed nine well-performing *Schisandra* resources from the *S. chinensis* resource bank and compared their lignan component contents. ZJ19 and ZJ27 emerged as resources with high lignan content, contributing to the advancement of superior *Schisandra* breeding. The lignan components in *Schisandra* fruits were ranked as follows: Schisandrol A > Schisandrin B > Schisandrin A > Schisandrin C > Schisandrol B > Schisandrol C. This ranking aligns with the findings of Liang Shuang [49,50,51], confirming that Schisandrol A is the predominant lignan component.

There were differences in the abundance of microorganisms among various samples. Based on the rhizosphere microbial abundance, the nine Schisandra resources were ranked as follows: ZJ22 > ZJ25 > ZJ19 > ZJ23 > ZJ24 > ZJ27 > ZJ26 > ZJ21 > ZJ20. Conversely, based on total lignan content, the ranking was as follows: ZJ27 > ZJ19 > ZJ20 > ZJ21 > ZJ24 > ZJ26 > ZJ22 > ZJ23 > ZJ25. We found that microbial abundance was negatively correlated with lignan content. This finding contrasts with the conclusions of Mu Maojun et al., who observed that a richer bacterial community in the rhizosphere of *Polygonatum cyrtonema* was associated with higher saponin and total alkaloid content [52]. Similarly, research by Junjie Tang et al. [53] indicated that a richer rhizosphere microbiome correlated with higher levels of active ingredients, such as pseudoginsenoside and ginsenoside in *Atractylodes macrocephala*. They suggested that this result might be related to the increased abundance of specific microorganisms such as *Streptomyces*, *Candida*, and *Frankia*. In contrast, the negative correlation between rhizosphere microbial abundance and lignan content in *Schisandra* may be attributed to significant differences in Schisandrol B and Schisandrin B content among the nine *Schisandra* resources. Both Schisandrol B and Schisandrin B are highly negatively correlated with rhizosphere microbial abundance, which may explain why total lignan content decreases as rhizosphere microbial abundance increases.

Many studies have indicated that the components used in Chinese herbal medicines are specifically related to rhizosphere microorganisms [54]. For example, *Stenotrophomonas* in the rhizosphere microorganisms of *Astragalus* promotes the accumulation of astragaloside [55]. Similarly, norank_f_*Anaerolineaceae*, norank_ f_*AKYG1722*, and norank_o_*Gaiellales* of *Lycium bacbarum* promoted the accumulation of betaine and polysaccharide [56], while *Bacillus amyloliquefaciens* in *Panax ginseng* promoted yield accumulation [57]. In our research, *Candidatus_Soilbacter* and *Bryobacter* exhibited a highly significant negative correlation with Schisandrol B. Both *Candidatus_Solibacter* and *Bryobacter* belong to the phylum *Actinobacteriota*. Studies have shown that *Acidobacteria* are Gram-negative acidophilus bacteria, possess a comprehensive set of physiological functional genes. These genes are involved in the carbon, nitrogen, and sulfur cycles, of they are genes for degrading polysaccharides, genes encoding transporters, and genes regulating the synthesis of secondary metabolites [58,59,60,61]. Therefore, it is possible that *Candidatus_Soilbacter* and *Bryobacter* play a role in regulating lignan synthesis.

Many factors affect rhizosphere microbial diversity. Pan Z., et al. demonstrated that the composition of rhizosphere microorganisms in *Schisandra* is closely related to soil fertility and moisture content [62]. Tong B., et al. found that soil type influences the community structure of rhizosphere microorganisms. These microorganisms are correlated with the active ingredients in *Schisandra*, providing new directions for further research on cultivation techniques to improve the quality and efficiency of *Schisandra* [63]. Li G. discovered that the rhizosphere microbial community structure of *Mongolian astragalus* varies in different growth stages, and applying microbial fertilizer during critical growth periods can enhance the growth of *Mongolian astragalus* [64]. Zhou N., et al. reviewed the relationship between rhizosphere microorganisms and the growth and alkaloid accumulation of *Polygonatum cyrtonema* [65]. They found that an increase in planting years leads to a decrease in rhizosphere microorganisms, resulting in the accumulation of diseases and a decline in the quality of *Polygonatum cyrtonema* [65]. Similarly, Jiang L., et al. observed that with increased planting years, the diversity of rhizosphere soil fungi of *Schisandra* decreases, providing new evidence for the study of *Schisandra* cultivation techniques [66].

## 5. Conclusions

In this study, ZJ19 and ZJ27 were identified as the *Schisandra* resources with the highest lignan content out of nine different resources. The rhizosphere soil bacteria *Candidatus_Solibacter*, *Bryobacter*, and *Gaiella* were found to have significant correlations with lignan content. These findings provide a basis for breeding superior *Schisandra* varieties, understanding the interaction between rhizosphere microorganisms and *Schisandra* affecting the synthesis of medicinal components, and developing *Schisandra*-specific microbial fertilizers.

## Figures and Tables

**Table 1:** Soil characteristics of *S. chinensis* resource bank.

pH	Organic Matter (OM)	Total Phosphorus (TP)	Total Potassium (TK)	Total Nitrogen (TN)	Alkaline Hydrolysable Nitrogen (AN)	Available Phosphorus (AP)	Available Potassium (AK)
5.31	3.27%	807.06 mg/kg	18,441.81 mg/kg	1922.6 mg/kg	164.02 mg/kg	142.57 mg/kg	114.82 mg/kg

**Table 2:** High-performance liquid gradient elution condition.

Time (t/s)	Velocity of Flow (mL/min)	A (%)	B (%)
0	1.0	45	55
20	1.0	25	75
40	1.0	22	78
45	1.0	22	78
47	1.0	5	95
52	1.0	5	95
55	1.0	45	55
60	1.0	45	55

**Table 3:** Statistics of *S. chinensis* fruit characteristics.

Sample ID	Length of Fruit Bunch (cm)	Stalk Length of Fruit Bunch (cm)	Number of Fruits per Fruit Bunch	Weight of Fruit Bunch (g)	Width of Fruit (mm)	Length of Fruit (mm)	Fresh Weight of Fruit (g)	Dry Weight of Fruit (g)
ZJ19	6.900 ± 0.964 b	2.567 ± 0.058 d	21.0 ± 2.646 bc	21.140 ± 5.142 abc	10.633 ± 0.850 c	10.533 ± 0.351 abc	0.983 ± 0.121 a	0.220 ± 0.026 a
ZJ20	9.167 ± 0.896 ab	3.233 ± 0.551 cd	25.0 ± 5.568 abc	24.753 ± 5.973 abc	12.033 ± 2.201 abc	11.533 ± 1.419 abc	0.993 ± 0.107 a	0223 ± 0.038 a
ZJ21	11.800 ± 4.190 a	3.833 ± 1.716 bcd	29.3 ± 12.055 ab	26.527 ± 8.135 ab	13.700 ± 0.947 ab	12.223 ± 0.127 ab	1.012 ± 0.128 a	0.227 ± 0.025 a
ZJ22	9.033 ± 1.966 ab	3.200 ± 0.346 cd	26.3 ± 1.528 abc	20.800 ± 5.888 abcd	12.813 ± 1.261 ab	10.857 ± 1.231 abc	0.844 ± 0.024 abc	0.153 ± 0.006 bc
ZJ23	7.867 ± 0.902 b	2.867 ± 0.153 cd	20.0 ± 5.196 bc	15.247 ± 5.342 cd	13.317 ± 1.136 ab	11.323 ± 1.181 abc	0.780 ± 0.091 bc	0.190 ± 0.020 ab
ZJ24	12.433 ± 1.102 a	7.900 ± 0.346 a	18.7 ± 5.508 c	17.030 ± 5.434 bcd	14.017 ± 0.594 a	12.383 ± 1.515 a	0.994 ± 0.046 a	0.167 ± 0.012 bc
ZJ25	12.033 ± 0.950 a	5.333 ± 1.531 b	24.7 ± 2.082 abc	20.373 ± 7.070 abcd	10.650 ± 0.490 c	10.227 ± 0.816 bc	0.875 ± 0.085 abc	0.157 ± 0.021 bc
ZJ26	10.067 ± 1.106 ab	4.433 ± 1.436 bc	19.0 ± 3.606 bc	10.453 ± 0.739 d	11.783 ± 0.667 bc	9.907 ± 0.895 c	0.723 ± 0.118 c	0.133 ± 0.021 c
ZJ27	9.600 ± 1.253 ab	2.933 ± 0.611 cd	32.0 ± 2.646 a	29.467 ± 1.559 a	12.080 ± 0.806 abc	10.940 ± 0.985 abc	0.928 ± 0.088 ab	0.170 ± 0.020 bc
Mean	9.878	4.033	24	20.643	12.336	11.103	0.904	0.182
RSD	4.61%	8.65%	5.14%	6.78%	2.33%	2.06%	2.66%	3.85%

Note: Different letters in this table indicate significant differences between samples, *p* < 0.05.

**Table 4:** Content of Schisandra lignan components in fruits of different *Schisandra* resources.

Sample ID	Schisandrol A (mg/g)	Schisandrol B (mg/g)	Schisantherin A (mg/g)	Schisandrin A (mg/g)	Schisandrin B (mg/g)	Schisandrin C (mg/g)	Total Lignans (mg/g)
ZJ19	5.730 ± 0.053 bcd	1.609 ± 0.120 b	0.919 ± 0.085 e	1.380 ± 0.196 cd	3.861 ± 0.269 a	0.342 ± 0.016 b	13.841 ± 0423 a
ZJ20	5.730 ± 0.234 bcd	1.884 ± 0.148 a	0.698 ± 0.068 f	1.098 ± 0.144 ef	2.789 ± 0.197 b	0.419 ± 0.028 a	12.619 ± 0.328 b
ZJ21	6.014 ± 0.133 b	0.402 ± 0.027 e	2.091 ± 0.050 a	1.466 ± 0.020 c	2.208 ± 0.039 bc	0.0803 ± 0.002 g	12.261 ± 0.143 b
ZJ22	5.626 ± 0.056 cd	1.104 ± 0.055 c	1.029 ± 0.008 d	1.202 ± 0.004 de	1.126 ± 0.013 e	0.062 ± 0.004 g	10.149 ± 0.015 d
ZJ23	5.510 ± 0.15 d	0.390 ± 0.029 e	0.597 ± 0.059 g	1.360 ± 0.185 cd	1.725 ± 0.115 cde	0.258 ± 0.013 c	9.839 ± 3.204 d
ZJ24	5.536 ± 0.317 d	1.550 ± 0.116 b	0.876 ± 0.083 e	1.130 ± 0.168 e	1.235 ± 1.084 e	0.219 ± 0.017 d	11.430 ± 0.481 c
ZJ25	5.133 ± 0.016 e	0.773 ± 0.036 d	0.737 ± 0.010 f	0.888 ± 0.015 f	1.954 ± 0.031 cd	0.242 ± 0.004 cd	9.726 ± 0.036 d
ZJ26	5.927 ± 0.074 bc	0.361 ± 0.046 e	1.131 ± 0.004 c	2.034 ± 0.035 b	1.398 ± 0.007 de	0.169 ± 0.003 e	11.020 ± 0.041 c
ZJ27	6.345 ± 0.270 a	0.707 ± 0.093 d	1.597 ± 0.051 b	2.959 ± 0.116 a	2.315 ± 0.060 bc	0.107 ± 0.004 f	14.031 ± 0.336 a
RSD	6.33%	58.06%	43.30%	41.11%	36.62%	54.97%	14.00%

Note: Different letters in this table indicate significant differences between samples, *p* < 0.05.

**Table 5:** Microbial α diversity in the rhizosphere of *S. chinensis*.

Sample ID	Shannon	Chao	Coverage
ZJ19	6.95 ± 0.04 a	4985 ± 142.2 b	0.96 ± 0.001 bc
ZJ20	6.37 ± 0.31 c	3925 ± 288.6 c	0.97 ± 0.002 a
ZJ21	6.54 ± 0.02 bc	3952 ± 75.97 c	0.97 ± 0.001 a
ZJ22	6.89 ± 0.09 ab	5789 ± 71.82 a	0.96 ± 0.0003 d
ZJ23	6.72 ± 0.23 abc	4684 ± 450.6 b	0.96 ± 0.003 bc
ZJ24	6.58 ± 0.11 bc	4551 ± 364.6 b	0.96 ± 0.003 bc
ZJ25	6.56 ± 0.06 bc	4954 ± 50.08 b	0.96 ± 0.0003 c
ZJ26	6.67 ± 0.09 abc	4467 ± 210.9 b	0.97 ± 0.001 b
ZJ27	6.50 ± 0.07 c	4531 ± 72.63 b	0.97 ± 0.001 bc

Note: Different letters in this table indicate significant differences between samples, *p* < 0.05.

## Data Availability

16SrDNA sequencing raw data can be downloaded at NCBI (http://www.ncbi.nlm.nih.gov/bioproject/1126850) (accessed on 31 May 2024). when the article is published. The data presented in this study are available on request from the corresponding author.
